# Common and uncommon audio-vestibular findings in COVID-19 patients

**DOI:** 10.1186/s43163-022-00308-9

**Published:** 2022-09-05

**Authors:** Ebtessam Hamed Nada, Amani Mohamed El-Gharib, Mahmoud Mandour

**Affiliations:** 1 Audiology Unit, ENT Department, Faculty of Medicine, Zagazig University, Zagazig, Egypt; 2 Audiology Unit, ENT Department, Faculty of Medicine, Tanta University, Tanta, Egypt; 3 ENT Department, Faculty of Medicine, Tanta University, Tanta, Egypt

**Keywords:** COVID-19, SSNHL, Hearing loss, Vertigo, BPPV, Labyrinthitis, Vestibular neuritis

## Abstract

**Background:**

COVID-19 is the new version of the old coronavirus known since 1960, which caused the Middle East respiratory syndrome (MERS-CoV) in 2012 and the severe acute respiratory syndrome (SARS) in 2003.

Symptoms included fever and cough, diarrhea and vomiting, and neurological symptoms like anosmia.

**Methods:**

One hundred twenty-eight patients diagnosed as COVID-19 with audio-vestibular complaints were subjected to audio-vestibular assessment and were included in the study.

**Results:**

In our study on COVID-19 patients who reported audio-vestibular complaints, hearing loss was found in 43.8% of patients in comparison to vertigo that represented 40.6% of cases. The most common type was sensorineural hearing loss representing 29.7% of patients and which was unilateral and sudden in 35.7% of them. Less commonly conductive hearing loss (CHL) was found in 14.1% of cases the most common form was bilateral mild to moderate CHL (83.3%) due to bilateral middle ear effusion.

Among cases with vertigo, the most common etiology was benign paroxysmal positional vertigo (BPPV) (42.5%) then uncompensated vestibular neuritis (VN) (31.5%), and lastly, combined BPPV with VN (25%) of cases. Less frequently we found tinnitus in (13.3%) which was bilateral in (64.7%), labyrinthitis (5.5%), and acute VN 5.5%).

The significant increase in the number of audiovestibular complaining cases that were observed in the course of the recurrent waves’ peaks pushed us to study the relationship between the pandemic and the audiovestibular system. The effect of COVID on AV systems is well noticed and management would be mandatory.

## Background

COVID-19 is the new severe acute respiratory syndrome coronavirus 2 (SARS-CoV-2) and is the most recently identified version of the old coronavirus known since 1960, which caused the Middle East respiratory syndrome (MERS-CoV) in 2012 and the severe acute respiratory syndrome (SARS) in 2003.

The recent pandemic of coronavirus emerged following a cluster of cases of pneumonia in the People’s Republic of China [1]. The very first outbreak started in early December 2019 in the Hubei Province and its capital Wuhan and resulted in a global pandemic.

The condition started with a limited human-to-human transmission seen mainly within families and the World Health Organization (WHO) announced this on January 22, 2020. On the 23rd of January, it was announced that the outbreak constituted a public health emergency of international concern [2]. Virus spread was recorded worldwide and was announced as a pandemic by WHO in March 11, 2020 [3]. Global spread included Egypt, and the first case was recorded in Egypt on February 14, 2020 [4].

By the time we write this paper the number of patients confined to this disease has reached 215,900,900 patients in more than 223 countries, and the number who died is over 4,494,855 (up to 27 August 2021). With about 193,050,345 patients recovered from the coronavirus infection according to the worldometer COVID-19 coronavirus pandemic [5].

The respiratory system is the main system affected in SARS-CoV-2, with multiple infiltrates of the lungs clinical picture includes shortness of breath, tachypnea, decreased oxygen saturation, and elevated C-reactive protein (CRP) [6].

Similarly, the cardiovascular system is involved in COVID-19 infection. The inflammation of the vascular system results in some changes (1) diffuse microangiopathic thrombi, (2) inflammation of cardiac muscle (myocarditis), and (3) cardiac arrhythmias, heart failure, and acute coronary syndrome [7, 8].

The lymphocytopenia characterizing the infection would disturb the innate and acquired immune responses resulting in delayed viral clearance, and hyper-stimulated macrophages and neutrophils [9].

The reported gastrointestinal manifestations of COVID-19 include diarrhea, nausea, vomiting, and abdominal pain [10]. SARS-CoV-2 also causes liver injury manifested by elevated serum alanine aminotransferase (ALT) and aspartate aminotransferase (AST) [11]. In most cases, the liver injury was transient and mild. However, severe liver dysfunction/injury has been reported in patients with severe disease [12].

There is clinical evidence that the SARS-CoV-19 has potential neuropathic properties. Several neurologic-related symptoms have been reported including headaches, dizziness, seizure, decreased level of consciousness, acute hemorrhagic necrotizing encephalopathy [13], agitation, and confusion.

Similarly, hearing loss has been reported in symptomatic and asymptomatic COVID-19 patients who were presented with SNHL whereas other cases had conductive hearing loss. Other symptoms reported with COVID were tinnitus and dizziness despite being less frequent [14]. On the other hand, in the same study by Soylemez, E., and Ertuguul, S. they stated that the vestibular compensation mechanism may hide vestibular symptoms and subsequently they recommended vestibular evaluation to be done with objective tests in these patients [15].

Now, it is strongly believed that any unexplained symptom affecting any body system may be attributed to COVID-19. We in our practice have noticed a significant increase in the rate of some audio-vestibular complaints in comparison to those before the pandemic, so we aimed at the descriptive analysis of the common audio-vestibular complaints encountered among patients with CVOID.

## Methods

One hundred twenty-eight adult cases (70 males and 58 females with age range, 20–60) that were diagnosed with coronavirus using any diagnostic tool for coronavirus were included in the study. They were examined in an audio-vestibular clinic in the time period since the declaration of the first wave in Egypt till now. Apart from cases presented with acute vertigo (confirmed COVID later on by progression of chest symptoms and investigations) all cases were presented within 3 to 12 months following infection).

Patients diagnosed with non-coronavirus infections were excluded. Also, patients with a history of old hearing loss, vertigo of any etiology, and those with an active middle ear infection (chronic suppurative otitis media) were excluded.

The patient presented for audiological or audio-vestibular evaluation based on the main complaint whether hearing loss, tinnitus, and/or vertigo.

All cases were referred from specialties like otorhinolaryngology, internal medicine, chest, and neurology.

### COVID diagnosis

Diagnosis of COVID was done by referral doctor (typical symptoms and investigations; computerized topography (CT) and laboratory investigations or polymerase chain reaction (PCR)) and explored from the patient by history taking or from the referral letter from the referral doctor. Investigations used included:

- CT chest representing the common radiological appearance and laboratory investigations including the complete blood count (CBC), D-dimer, and CRP.
- PCR if possible for some cases.

All cases were subjected to:

- Full history taking involving symptoms encountered throughout the course of the disease, medications used, and intensive care unit ( ICU) admission if any.
- For cases with a previous history of COVID, history of complications or other system affection was added.
- History of medical illness.
- Examination:
  - ▫ Audiological assessment:
    - ▪ Otoscopic examination.
    - ▪ Tympanometry.
    - ▪ Pure tone audiometry.
  - ▫ Vestibular examination:
    - ▪ Bedside vestibular testing.
    - ▪ Videonystagmography (VNG) testing.
  - ° A record of oxygen levels during the course of the disease is measured by a pulse oxymeter.
- Laboratory investigations including, D-dimer, CBC, CRP and PCR whenever possible (previously done under supervision of the referral doctor).

*All patients were* informed about the study and approved the study.

*The research was approved by* The Research Ethics Committee (Approval code No.9039).

### All patients were within the mild or moderate degree of the COVID

According to Parasher [16], patients were categorized into mild, moderate, or severe according to the symptoms on a presentation whereas mild-to-moderate cases are those presented with symptoms such as fever, persistent dry cough, body aches, and occasional breathlessness.

- Mild illness SpO_2_ levels of 94–97% in room air.
  - ° Fever, sore throat, dry cough, malaise, and body aches or
  - ° Nausea, vomiting, abdominal pain, and loose stools
- Moderate illness SpO2 levels of 90–94% in room air.
  - ° Symptoms of pneumonia (persistent fever and cough) without hypoxemia
  - ° Significant lesions on high-resolution CT chest.

## Results

One hundred twenty-eight patients were included in the study; all were COVID-19 patients diagnosed by their referring doctors.

The findings among COVID patients with audio-vestibular symptoms were as follows: 20.3% had hearing loss, 13.3% had tinnitus, 36.7% had vertigo (true spinning), and 6.25% of cases had dizziness. While 14.1% of cases had both hearing loss associated with ear block (hearing loss and own voice augmentation), 3.9% of cases had both hearing loss and vertigo in combination and 5.5% had hearing loss and tinnitus together (Table 1).

**Table 1:** Different complaints reported by COVID patients

Complaints	Character		*N*. (%)	Total (100%)
Hearing loss.	Sudden	Unilateral	20 (76.9%)	26 (20.3%)
		Bilateral	0 (0%)	
	Other	Unilateral	4 (15.4%)	
		Bilateral	2 (7.7%)	
Tinnitus.	Unilateral		11 (64.7%)	17 (13.3%)
	Bilateral		6 (35.3%)	
Vertigo.	Acute		7 (14.9%)	47 (36.7%)
	Other		40 (85.1%)	
Dizziness			8 (100%)	8 (6.25%)
Combined hearing loss and ear-block	Unilateral		3 (16.7%)	18 (14.1%)
	Bilateral		15 (83.3%)	
Combined hearing loss and vertigo			5 (100%)	5 (3.9%)
Combined hearing loss and tinnitus			7 (100%)	7 (5.5%)
Total	128 (100%)			

This table describes findings among COVID patients with audio-vestibular symptoms. 20.3% had hearing loss, 13.3% had tinnitus, 36.7% had vertigo (true spinning), and 6.25% of cases had dizziness. While 14.1% of cases had both hearing loss associated with ear block (hearing loss and own voice augmentation), 3.9% of cases had both hearing loss and vertigo in combination and 5.5% had hearing loss and tinnitus together

**Fig. 1:**
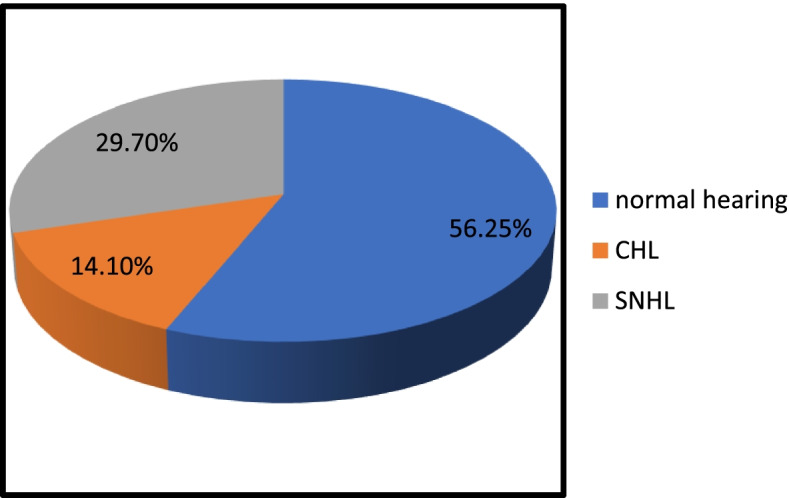
Audiological findings in COVID patients

As regards the type of hearing loss encountered we found that 56.25% of COVID cases involved in the study had normal hearing while 29.7% had SNHL and 14.1% had CHL (Fig. 1).

**Fig. 2:**
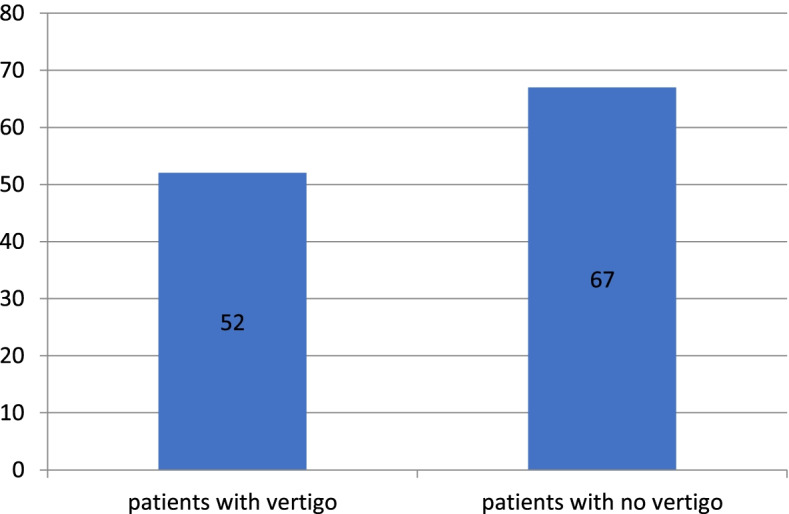
Vestibular complaint among COVID-19 patients

We found also that 40.6% of COVID patients with audio-vestibular complaints had vertigo either alone or in combination with hearing loss (Table 1 and Fig. 2).

**Fig. 3:**
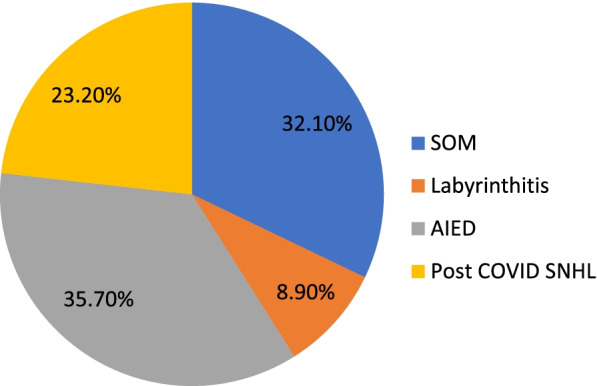
Audiological diagnosis according to history and audiological findings.

And as regards the diagnosis of the audio-vestibular complaint reported in COVID patients, we found middle ear effusion or secretory otitis media (SOM) based on tympanometry results, labyrinthitis as confirmed with SNHL of sudden onset associated with vertigo, autoimmune inner ear disease (AIED) presented by sudden SNHL (responsive to steroid treatment), and cases with SNHL developed gradually afterward (Fig. 3).

**Fig. 4:**
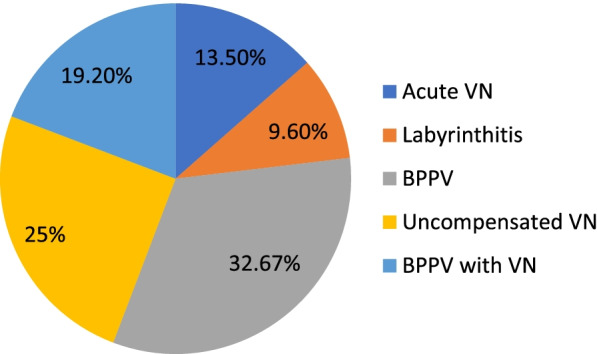
Vestibular diagnosis based on history, bedside, and VNG examination

Diagnostic presentation of cases with vertigo among COVID patients, stating 7 cases with acute vestibular neuritis as proved by vertigo, positive HINTs test, features of an acute peripheral vestibular lesion with normal hearing, and labyrinthitis as proved by the presence of SNHL with sudden onset of vertigo. BPPV was diagnosed with the Dix-Hallpike test. VNG and bedside features of uncompensation are there to diagnose this condition (Fig. 4).

## Discussion

Multisystem affection is reported during the COVID-19 pandemic. Systems involved include in primary basis respiratory system causing cough, fever, and increased respiratory rate [6], and the cardiovascular system, causing diffuse micro-angiopathic thrombi, myositis, and cardiac arrhythmia with complications that may cause death [7, 8].

Other systems affected include the gastrointestinal system causing diarrhea, nausea, vomiting, and abdominal pain [10]. Enormously, neurological manifestations more recently occurred and included anosmia (i.e., loss of the sense of smell) and ageusia (i.e., loss of taste) [17].

More recently, in our audio-vestibular practice, a significant increase in the rate of patients with sudden hearing loss, vertigo, and some other complaints was noticed indicating the possible effect of the ongoing pandemic on the audio-vestibular system. Moreover, audio-vestibular findings were reported in the literature in patients with COVID either asymptomatic or symptomatic.

Our study sample included 128 cases with audio-vestibular complaints referred to us from otorhinolaryngology and internal medicine.

All cases were diagnosed as COVID based on the presence of clinical evidence of COVID-19 infection, laboratory tests (lymphocytopenia, thrombocytopenia, elevated C-reactive protein, and erythrocyte sedimentation rate), CT scan of the chest [18] and PCR (in some cases who reported hospital admission) [19]. All investigations are interpreted in the association of the clinical picture and epidemiologic information to confirm the diagnosis [1].

Findings revealed hearing loss in 56 cases (43.8%) of which 20 cases (35.7%) presented with sudden onset of unilateral SNHL in the course of upper respiratory tract infection. No specific configuration was detected; all cases were referred for otorhinolaryngology.

Around 2/3rd of cases (13 cases) responded to the usual treatment of steroid therapy in the usual large dose used (1 mg/kg/day) with only 3 cases proceeding for intra-tympanic injection because of general contra-indication of systemic steroid therapy (uncontrolled diabetics, hypertension) or in conjunction with systemic steroids in case of absence of response to systemic therapy.

The pathophysiology of sudden SNHL in COVID patients may be attributed to viral infection in the course of the disease and being responsive to steroids recommends this etiology of viral infection or the auto-immune inner ear disease (AIED). Viral infections have an important place in the pathogenesis of some audio-vestibular diseases, and COVID as a viral infection affecting the respiratory system may have co-morbidity affecting the cochlea and labyrinth as a whole [20, 21].

On the other hand, COVID-19 is reportedly associated with Guillain Barre Syndrome (GBS) which is an acute immune-mediated disease with central and peripheral nerve manifestations [22]. This may explain immune problems that may lead to AIED if it is not the effect of any of the drugs being tried with COVID-19.

A similar finding was reported by other studies in which some patients with COVID-19 had acute unilateral SNHL hearing loss [23].

Inversely, the other 6 cases presented with hearing loss of recent onset following COVID which was unilateral in 4 cases and bilateral in 2 cases. No hearing loss was there before they had COVID. Presentation of cases was after the end of the COVID attack, most of the cases reported COVID within the past 6 months. No other etiology could be the cause of hearing loss as detected by careful history taking.

What if it was the effect of any of the drugs used, or the effect of the disease, we cannot tell. But the fact is that drugs used with COVID have been changing over the course of the newly emerging pandemic. For example, hydroxychloroquine and chloroquine have been prescribed for about 12% of COVID-19 patients in Europe [24]. These are antiviral medications with known adverse effects like tinnitus and hearing loss [25–27]. Afterwards, ivermectin (anthelmintics) was used as another line of treatment causing dizziness as a side effect as well [28]. So, it is the disorder that is to blame as it is the factor that is there all the time.

High-frequency hearing loss was reported by Mustafa in 2020 [29] and he justified his finding by that the virus affects the most sensitive area of the cochlea which is the basal 1/3^rd^ which represents the high-frequency region.

In a similar study, Kılıç et al. [30] stated that 20% of patients who came to the clinic with a diagnosis of sudden hearing loss in the Covid process had positive PCR test. And based on their finding they recommended that individuals, who come to the clinic with the diagnosis of sudden hearing loss in this process, should be evaluated in terms of COVID-19 even if they do not have a specific symptom.

Tinnitus without an associated hearing loss was reported in 17 patients (13.29%) of cases: 6 of them (35.3%) had bilateral tinnitus and 11 (64.7%) had unilateral tinnitus, all reported noise character of tinnitus, while only 2 patients reported pulsatile type of tinnitus. Tinnitus also was reported in 23.2% of patients in the study by Viola et al in 2021 [31]. While in another study by De Luca et al in 2021 fewer rate of tinnitus was reported but we can explain that by the fact that the paper was talking about sudden sensory neural hearing loss only not about any audio-vestibular findings that may be present in COVID-19 patients [32].

The poor general condition of the COVID patients especially with the haphazard use of antibiotics and different treatment protocols and the poor appetite of patients during the course of the disease may be the cause of poor vascularization of the inner ear and the resulting tinnitus. Patients with unilateral tinnitus may have developed tinnitus in the other ear if the duration of the attack was prolonged.

In their study, Lechien et al. [24] studied the symptoms of 1420 COVID-19 patients and reported tinnitus in 5 (3.5%) of these patients. And according to Almufamij et al. [33], tinnitus was reported in 4 studies conducted to evaluate the effect of COVID on the audio-vestibular system: Lecchien et al. [24] was one of them and Cui et al. [34], Fidan [35], and Sun et al. [36] were the other studies.

Whereas the other 18 cases (14.1%), presented with ear block, revealed middle ear effusion (MEE) which was unilateral in 3 cases (16.7%) and bilateral in 15 cases and was attributed to the upper respiratory tract infection associated with the condition, the thick mucus and irritation of the mucosal membranes. Most of the cases had bilateral SOM, and less frequently, unilateral SOM was detected.

Fidan [35] reported otalgia, tinnitus, and unilateral conductive hearing loss in a patient with COVID-19.

On the other hand, the more obvious symptom encountered with COVID was vertigo, 52 cases of COVID (40.6%) reported some type of vertigo, where 7 cases of them (13.5.%) had vertigo as the prominent symptom at the very beginning of the COVID attack (other symptoms developed later or not developed at all, i.e., vertigo was the sole manifestation where COVID was suspected and confirmed by laboratory investigations) and 5 cases (9.6%) had their vertigo associated with SNHL denoting labyrinthitis as a provisional diagnosis confirmed later on by free CPA (cerebello-pontine angle) radiology.

The majority of cases (40 cases, 76.9%) presented with vertigo, dizziness, and imbalance with a history of COVID within the past 4 to 6 months before the complaint.

For cases with acute vertigo, the HINTS test was done as usual to differentiate acute vestibular neuritis from acute stroke. All cases showed spontaneous nystagmus that was unidirectional and increased in frequency on looking to the fast phase (Alexander’s low) with intact fixation suppression (nystagmus decreased in amplitude not disappearing completely). All cases were instructed to follow treatment for acute vertigo with the protocol followed for COVID once laboratory investigations were confirming COVID. Similar findings of acute vertigo were reported as few as 5.9% in the study done by Viola et al. in 2021 which was a multicentric study including 15 Italian hospitals [31].

Those 40 cases were diagnosed as follows: 17 cases (42.5%) had BPPV, 13 cases (32.5%) had an uncompensated peripheral vestibular lesion, and 10 (25%) cases had both BPPV and VN.

For chronic patients, vestibular bedside testing was performed together with VNG testing. Findings were as follows: 17 cases (42.5%) had BPPV (mostly posterior canal, and 2 cases had bilateral posterior canal BPPV); 13 cases (32.5%) had uncompensated peripheral vestibular lesion as documented by positional nystagmus, spontaneous nystagmus in few cases, and unilateral caloric weakness on top; and 10 (25%) cases had both problems found in which we concluded BPPV on top of vestibular neuritis.

Usually, vestibular neuritis may develop after an upper respiratory tract infection causing severe vertigo in affected patients [20]. Viral infections have an important place in the pathogenesis of many audio-vestibular diseases [20, 21]. Moreover, viral labyrinthitis in particular may affect hearing and balance. It may also seriously damage brain functions.

Among the etiologies available for vestibular neuritis, it is believed to be an inflammatory disorder of viral origin affecting the vestibular portion of the eighth cranial nerve selectively [37]. Other causes reported for the development of VN are vascular etiology and immunologic etiology [38]. All are applicable within the process of COVID-19 infection. It has been reported that coronaviruses have neuro-trophic and neuro-invasive characters [39]. Therefore, coronaviruses may cause peripheral neuropathies.

Furthermore, although most cases of BPPV are idiopathic in origin, mostly result from degeneration of the macula. Secondary causes of BPPV refer to identifiable causes of otoconial dislodgement. These include otologic and nonotologic surgery, head trauma, or any means by which a sufficient mechanical force reaches the inner ear [14, 40, 41]. Forced cough in a patient with COVID and for a considerable period of time may be the cause of trauma reaching the inner ear.

From another point of view, the overlap between VN and BPPV is reported as the disease can develop via inner ear disorders which ultimately lead to the degradation and disassociation of otoconia from their native gelatinous substrate. These include vestibular neuritis, Meniere’s disease, and sudden sensorineural hearing loss (SNHL) [42, 43].

Management of all cases implemented the use of vestibular rehabilitation therapy (VRT) which was customized according to the case. Measures included repositioning maneuvers, gaze stabilization exercises, and optokinetic (OPK) training for a patient with visual vertigo.

Vestibular compensation mechanism may hide vestibular symptoms in peripheral vestibular disorder patients accordingly; vestibular evaluation should be done with objective tests in these patients [15].

Worth mentioning is that is it safe for audiovestibular practice during panndemic? [44]. Actually, it was possible to perform audio-vestibular tests during the COVID-19 pandemic by using necessary protective equipment (protective masks and face shields) and disinfecting the potential surfaces (including headphones using suitable sterilizers), and keeping time period between patients in the cabins permitting aeration and air clearance.

Vomiting during vestibular tests, uncovering the nose and mouth for lip reading, and small-sized test cabins are the main risk factors of contamination in the audiology clinics.

World Health Organization (WHO) and national guides could be used for general considerations [45]. But the audiology associations guidelines specific for the audiology clinics are also very helpful [46, 47].

Audio-vestibular problems as a result of COVID are neither infrequent nor unexpected, as COVID is mainly a respiratory problem affecting airways and vestibular neuritis is usually a consequence of upper respiratory tract infection. Moreover, immune modulation occurs with COVID either in the course of the disease or as a result of its treatment and may be the cause of inner ear affection with subsequent hearing loss and vestibular problems.

## Conclusions

After the extent of the new pandemic of COVID-19, most of the studies concentrated on the life-threatening effects of it as the spread of infection and mortality rate were forcing the world for social distancing with their effect on the world economy. With the emergence of more and more mutants of the virus, more systems affection was reported. SSNHL, SNHL, CHL, and vertigo of vestibular neuritis origin and BPPV were reported and managed. SSNHL, AIED, and CHL were found in our study group, in addition to BPPV, VN, and tinnitus.

We suggest that COVID patients would proceed for audio-vestibular assessment after stabilization of their condition to give access to early diagnosis and management.

## Data Availability

The datasets used and/or analyzed during the current study are available from the corresponding author on reasonable request.
